# The Prevalence of Burnout and Its Associated Factors Among Surgical Specialists in Kuwait Ministry of Health Hospitals

**DOI:** 10.3389/fpubh.2022.679834

**Published:** 2022-01-31

**Authors:** Amr Akl, Idrees Mohiyaldeen, Rashed Alshatti, Omer Alenezi, Ryan Dougherty, Abdullah Al-Raihan, Salman Alotaibi, Nardine Tadros, Joseph C. Longenecker

**Affiliations:** ^1^Kuwait Ministry of Health, Rotating Internship Program, Kuwait City, Kuwait; ^2^Faculty of Medicine, Department of Community Medicine and Behavioural Sciences, Kuwait University, Kuwait City, Kuwait; ^3^Department of Quality and Accreditation, Kuwait Ministry of Health, Kuwait City, Kuwait; ^4^Faculty of Public Health, Kuwait University, Kuwait City, Kuwait

**Keywords:** Kuwait, surgery, surgeon, burnout, prevalence

## Abstract

**Background:**

Workplace burnout is a state of emotional exhaustion (EE), depersonalization of others (DP), and low personal accomplishment (PA) owing to workplace stressors. This study aimed to assess the prevalence of burnout and its associated factors among surgical specialists in Kuwait.

**Methods:**

This cross-sectional study assessed the prevalence of burnout among 489 surgeons in practice at Kuwait Ministry of Health (MOH). Data were collected using a self-administered questionnaire. Burnout was assessed using the Maslach Burnout Inventory, which defines burnout as having a high score of EE or DP, or a low score of PA; with severe burnout involving all. The associations between burnout or severe burnout with sociodemographic and occupational characteristics were assessed using multivariate binary logistic regression to adjust for potential confounders.

**Results:**

Prevalence estimates for burnout and severe burnout were 76.9% (95% Confidence Interval = 72.9–80.7%) and 14.0% (11.0–17.5%), respectively. The prevalence estimates for high EE, high DP, and low PA scores were 44.7, 43.1, and 47.2%, respectively. The prevalence of burnout and severe burnout was highest among neurosurgeons. Burnout was lowest among otolaryngology surgeons and severe burnout was lowest among cardiothoracic surgeons. After adjustment, burnout was associated with younger age, lower income, and more on-calls per month. The four most common sources of stress included less family time, verbal assault, case overload, and highly complicated cases.

**Conclusion:**

The prevalence of burnout among surgeons in Kuwait MOH hospitals is high. Occupational health programs should use these findings to design and implement interventions that can treat and prevent burnout in this population.

## Key Message

What is already known about this subject?

Studies have shown that occupational burnout among medical specialties is associated with job dissatisfaction and has a negative impact on health and job performance. It is characterized by three main areas: emotional exhaustion, depersonalization, and reduced personal achievement. These have been found to be prevalent in all medical specialties, with a higher than average prevalence among surgical specialties.

What are the new findings?

The prevalence of burnout among surgeons in Kuwait was found to be 76.9%, with the common sources of stress being poor work-life balance, case overload, verbal assault from patients, assignment of high-risk cases, failure to get deserved promotions, and malpractice lawsuits. Specialists in neurosurgery, general surgery, urology and orthopedics were found to have very high levels of burnout (>75%) when compared to other surgical specialties.

How might it impact clinical practice in the foreseeable future?

These results may be used to inform multidisciplinary policies and programs to address the underlying causes of high levels of burnout among various surgical specialties, potentially enhancing surgeons' quality of life and job performance, as well as improving the quality of patient care.

## Introduction

Workplace burnout is defined as a state of emotional exhaustion (EE), depersonalization (DP), and low sense of personal accomplishment (PA) from prolonged exposure to demanding work situations (1), and has been reported as highly prevalent among physicians around the world (2–7). Burnout in this population can result in negative consequences both to the physician and the patient. The personal and occupational health implications of workplace burnout for the physician include symptoms of moodiness, depression, sleep disturbances and loss of interest in occupational or personal life activities (5). These health effects in turn may also interfere with the physician's ability to provide quality healthcare by impairing clinical judgement (8). Physician burnout can additionally result in dehumanizing patient care, false diagnoses, preventable medical errors, absenteeism, and withdrawal from the profession (2, 5). It is important to study burnout in the physician population due to these occupational and healthcare quality issues. Ministries of Health, Occupational Health departments, and hospital administrators can use such data to improve programs for physicians and to develop occupational health policies and prevention programs that aim to decrease the effects of burnout and improve patient care.

The Maslach Burnout Inventory (MBI) is the most commonly used tool to assess burnout, being composed of subscales for each of the three components of burnout (EE, DP, and low PA) (9). Burnout has been assessed using the MBI among practicing physicians in many countries with wide differences in the prevalence, ranging for example, from an estimated prevalence of 19% in Japan and 54.4% in the United States (10–12). When comparing burnout prevalence between studies, it is important to note the definition of “burnout” used. Some studies define burnout as an extreme score in any one of the three MBI axes (EE, DP, or PA) (13); whereas other studies define it as a high score in either EE or DP only (11).

Several studies of burnout among physicians have been conducted in the Middle Eastern region (2, 6, 7, 10, 14). A recent review of seven studies in Arab countries assessed the prevalence of burnout among physicians reported ranges of burnout from 12.6% in Qatar up to 70% in Saudi Arabia (6). However, few previous studies have assessed the differences in burnout prevalence between the surgical specialties, or assessed the prevalence of severe burnout, as defined as having an extreme score in all three components (5, 8).

Investigating factors associated with surgeon burnout is needed to identify subgroups at risk, to plan and implement targeted interventions to prevent the condition, and to improve our understanding of this psychological epidemic and how to best address it. Studies have found associations between burnout and work-life imbalance (2, 5, 11, 15), working longer hours (5, 8), being part of a residency program (5, 13), lack of social support (16), and medical errors (17). A systematic review of more than 100 articles found multiple factors associated with burnout that can be used to guide policies and interventions to reduce burnout in the workplace (16).

To our knowledge, with the exception of one study on burnout among surgical residents (7), the prevalence of burnout and its associated factors among practicing physicians and, more specifically, surgeons, has not previously been reported in Kuwait. This study aims to assess the prevalence and associated factors of burnout among surgeons in Kuwait.

## Methods

### Population, Study Design, and Study Procedures

The target population of this cross-sectional study included all currently active surgeons (*n* ≈ 916) in all five secondary and ten tertiary Ministry of Health hospitals in Kuwait. Physicians from other specialties and interns who rotate between different specialties were excluded. Data collection teams visited all the hospitals mentioned above between December 31, 2017, and January 21, 2018. Of 489 surgeons approached, 445 (91%) agreed to participate and completed the questionnaire anonymously.

This study was approved by the Kuwait University Health Sciences Center Ethics Committee for Undergraduate Research and the Kuwait Ministry of Health Standing Ethics Committee for the Coordination of Medical and Health Research on 12/24/2017 (MOH ID: 812) with approval from the directors of each hospital before approaching surgeons. Written informed consent was provided by each surgeon after an explanation of the study procedures and before administering the self-administered study questionnaire.

Data collection teams comprised of two investigators, both of whom approached surgeons in their workplace on a basis of convenience during the data collection period. Respondents were requested not to confer with others while completing the questionnaire. The 53-item questionnaire, based on the MBI (8) was administered in the English language only, since English is the primary language used in medical practice in Kuwait. The questionnaire included sections regarding sociodemographic characteristics, occupational status, self-identified sources of stress, and burnout. The MBI is comprised of three domains, including depersonalization (DP, five items), emotional exhaustion (EE, nine items), and low personal accomplishment (low PA, eight items). The participant ranks a response to questions such as “How often do you experience the following:” followed by statements such as “I feel emotionally drained from my work,” using a seven-point Likert scale ranging from 0 (never) to 6 (daily). The score for each subscale is calculated by summing the values of all answers (9). The MBI has been validated in Western countries (sample size, 1,316) (9). Cronbach's alpha was used to assess internal consistency, with reliability coefficients of 0.90 for emotional exhaustion, 0.79 for depersonalization, and 0.71 for personal accomplishment (9). The test-retest reliability coefficients were 0.82 for emotional exhaustion, 0.6 for depersonalization, and 0.8 for personal accomplishment (9).

### Coding of Burnout Variables

Score cut-points for each of the burnout subscales have been empirically determined using standard normative tertiles of the MBI (9). Normative tertile scores for EE included “low EE” (sub-score 0-18), “average EE” (sub-score 19-26), and “high EE” (sub-score 27-54). Normative tertile scores for DP included “low DP” (sub-score 0-5), “average DP” (sub-score 6-9), and “high DP” (sub-score 10-30). Normative tertile scores for PA included “low PA” (sub-score 0-33), “average PA” (sub-score 34-39), and “high PA” (sub-score 40-48).

A score in the highest normative tertile for EE and DP and the lowest normative tertile for PA corresponded to a high level of burnout for that subscale. Following the method used by Elmore et al. (13), an overall “burnout” variable was defined as a score in the most extreme normative tertile of any of the three subscales (i.e., an EE score of 27 or higher, OR a DP score of 10 or higher, OR a PA score of 33 or lower); and “severe burnout” was defined as a score in the most extreme normative tertile of all three subscales (i.e., an EE score of 27 or higher AND a DP score of 10 or higher AND a PA score of 33 or lower). These were the binary variables used to assess associations of participant characteristics with burnout and severe burnout.

Another 4-category burnout variable based on the three subscales was defined as: 0 = No extreme tertile score, labeled “No Burnout”; 1 = One extreme tertile score, labeled “Mild Burnout”; 2 = Two extreme tertile scores, labeled “Moderate Burnout”; 3 = Three extreme tertile scores, labeled “Severe Burnout.”

For comparison purposes, we also coded an alternative definition of “burnout” used by some other studies as a score in the most extreme normative tertile of either the EE or DP subscales (i.e., an EE score of 27 or higher, OR a DP score of 10 or higher) (11).

### Statistical Analysis

All statistical analyses were conducted using SPSS version 25 (IBM Corporation, Statistics for Windows, Armonk, NY). Frequencies, percentages, means, standard deviations, medians, and interquartile ranges were used for descriptive analyses, as appropriate. Statistical significance for associations between categorical variables was tested using chi-square and Fisher's exact test, as appropriate. For ordinal variables showing a trend across ordered groups, we used the Chi-Square test for trend. For associations between continuous variables and categorical variables, statistical significance was tested using Student's *t*-test, Mann-Whitney *U*-test, ANOVA, and Kruskal-Wallis tests, as appropriate. All tests were two-sided and a *p*-value of <0.05 was considered significant. A four-point Likert scale was used to assess sources of stress, ranging from 1 (not stressful at all) to 4 (very stressful). For analysis purposes, this scale was reduced to two categories (“stressful/very stressful” vs. “not stressful/slightly stressful”).

Confounding was controlled using multivariable binary logistic regression models with either “burnout” or “severe burnout” (as described above) as dependent binary variables. In unadjusted analyses (see Supplementary Table 1 in the online appendix), nine sociodemographic or occupation-related covariates were significantly associated with either burnout or severe burnout: age, marital status, income, physician rank, general surgery (vs. all other specialties), years of career, annual weeks off, working hours/day, and on-calls per month. Age, rank, income, and years of career were generally co-linear variables, so we decided to use years of career as the primary variable to represent these four variables as a covariate in the multivariable models. We also entered as covariates into all models the occupation-related variables that were significantly associated with either burnout or severe burnout in the unadjusted analyses and that were stable in the models. The final list of adjustment variables therefore included: general surgery (vs. all other specialties combined), years of career, annual weeks off, and on-calls per month. For the age, income and rank models, years of career was removed from the models because of collinearity. All final burnout and severe burnout models demonstrated goodness-of-fit, as tested by the Hosmer-Lemeshow Test (all *p*-values not significant). The Omnibus test of model coefficients (comparing the −2 log-likelihoods of the base model and the new model) was significant for all final burnout and severe burnout models (all *p*-values significant).

## Results

The mean age of participants was 38.6 years, the vast majority were men, and approximately half were of Kuwaiti nationality (Table 1). The most common surgical specialty was general surgery (37.1%), and most participants had their training in Kuwait, Arab countries, or Asia.

**Table 1:** Socio-demographic and occupational characteristics of surgeons in Kuwait MOH hospitals, 2018.

**Characteristic**	**Frequency**	
	***n***	**(%)**
Mean age (±SD)	38.6	(±9.8)
Sex (% male)	388	(87.2)
**Nationality**		
Kuwaiti	212	(47.6)
Non-Kuwaiti Arab	197	(44.3)
Non-Kuwaiti Non-Arab	36	(8.1)
**Marital status**		
Single	96	(21.6)
Married	341	(76.6)
Divorced	8	(1.8)
**Monthly income (Kuwaiti Dinars)**	
<2,000	217	(48.7)
2,001-3,000	138	(31.0)
3,001-4,000	41	(9.2)
>4,000	49	(11.0)
**Smoking status**		
Never-smoker	285	(64.0)
Ex-smoker	42	(9.4)
Current smoker	118	(26.5)
Initiation before surgical career	94	(21.1)
Initiation during surgical career	24	(5.4)
**Surgical specialty**		
General surgery	165	(37.1)
Neurosurgery	12	(2.7)
Plastic surgery	15	(3.4)
Urology	50	(11.2)
Ophthalmologic surgery	40	(9.0)
Cardiothoracic surgery	14	(3.1)
Orthopedic surgery	78	(17.5)
ENT surgery	50	(11.2)
Oncology surgery	8	(1.8)
Transplant surgery	5	(1.1)
Vascular surgery	6	(1.3)
Maxillofacial surgery	2	(0.4)
**Surgical residency location**		
Kuwait	154	(36.8)
Arab countries and Asia	218	(52.0)
US/Canada/Australia/EU	47	(11.2)
Median duration of surgical career, years [IQR]	11	[5,17]
Mean working hours per day (±SD)	8.1	(±1.9)
Median teaching hours per week [IQR]	2	[0, 6]
Median on-calls per month [IQR]	7	[6,8]
Mean weeks of vacation/year (±SD)	4.18	(±1.6)
Practice in private sector (%)	64	(14.4)

A majority of participants identified the following factors as either “stressful” or “very stressful”: having less time to spend with family, being verbally assaulted, being overloaded with cases, being assigned a case with a high probability of complications, not getting deserved promotions, and receiving malpractice lawsuits (Table 2).

**Table 2:** Prevalence of self-identified sources of stress and associations with physician rank.

**Self-identified sources of stress**	**Proportion responding that the source of stress was “stressful” or “very stressful”**					
	**All**		**Assistant Reg/ registrar** **(***n*** = 277)**	**Senior registrar** **(***n*** = 71)**	**Specialist/ consultant** **(***n*** = 97)**	
	***n***	**(%)**	**(%)**	**(%)**	**(%)**	***P***-**value**
Having less time to spend with family	305	(68.7)	71.1	72.9	61.9	0.21
Verbal assault from patients or families	293	(66.0)	62.8	78.6	66.0	0.039
Being overloaded with cases	284	(64.0)	65.3	72.9	53.6	0.036
Assigned cases with high complication risk	279	(62.8)	63.2	67.1	58.8	0.59
Failure to get deserved promotions	269	(60.6)	61.0	71.4	51.5	0.045
Malpractice lawsuits filed against you	265	(59.7)	55.2	74.3	61.9	0.019
Residency exams	192	(43.2)	51.6	35.7	24.7	<0.001^a^
Telephone calls from patients	189	(42.6)	38.3	55.7	45.4	0.019
Miscommunication with colleagues	164	(36.9)	35.4	31.4	45.4	0.12
Being passed over for interesting cases	164	(36.9)	41.9	34.3	24.7	0.002^a^
Inadequate supervision by seniors	162	(36.5)	46.6	30.0	12.4	<0.001^a^
Not being assigned enough cases	134	(30.2)	30.7	27.1	30.9	0.80
Miscommunication with OR nurses	125	(28.2)	22.7	20.0	49.5	<0.001^a^

a *P-value for linear trend*.

**Figure 1:**
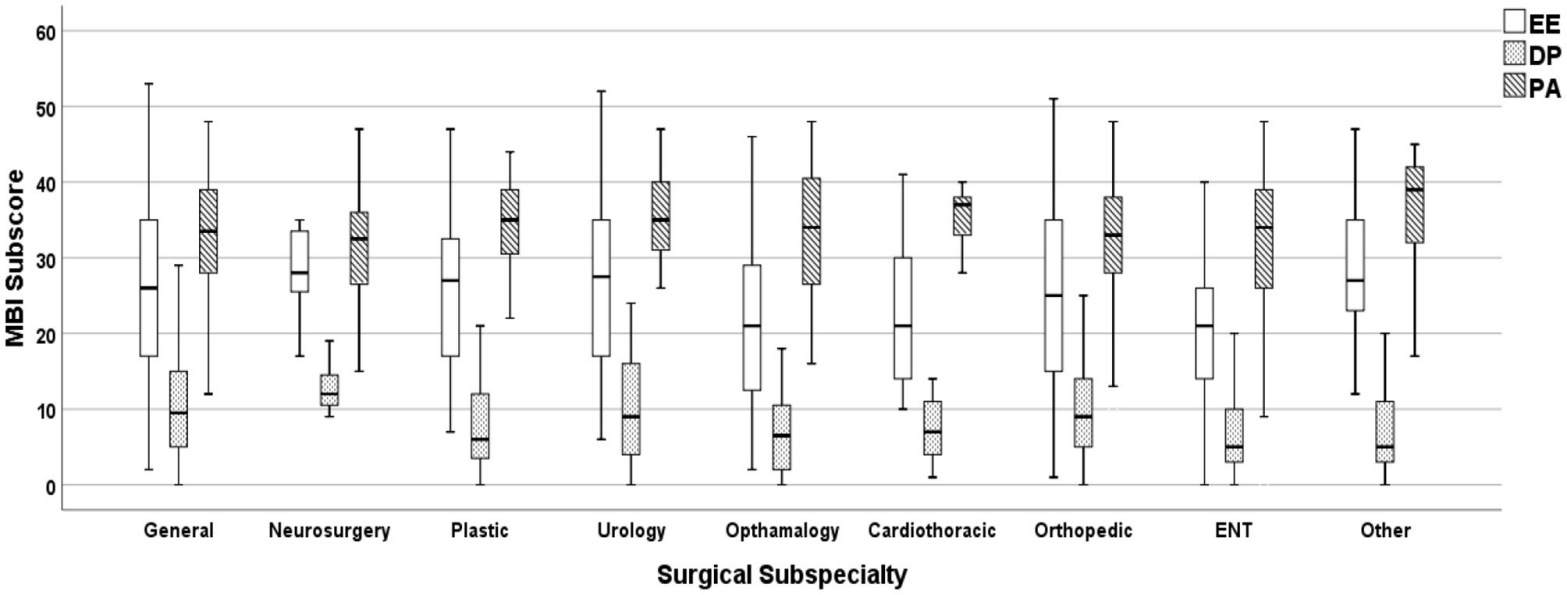
Distribution of MBI subscale scores, by surgical specialty.

The prevalence of the three indicators of burnout, according to the MBI were as follows: high EE score, 44.7%; high DP score, 43.1%; low PA score, 47.2%. In relation to surgical specialties, neurosurgeons had the worst median score for EE, DP, and PA. In contrast, ENT surgeons had relatively the best median score in EE and DP, while cardiothoracic surgeons had the best median score in PA (Figure 1).

**Figure 2:**
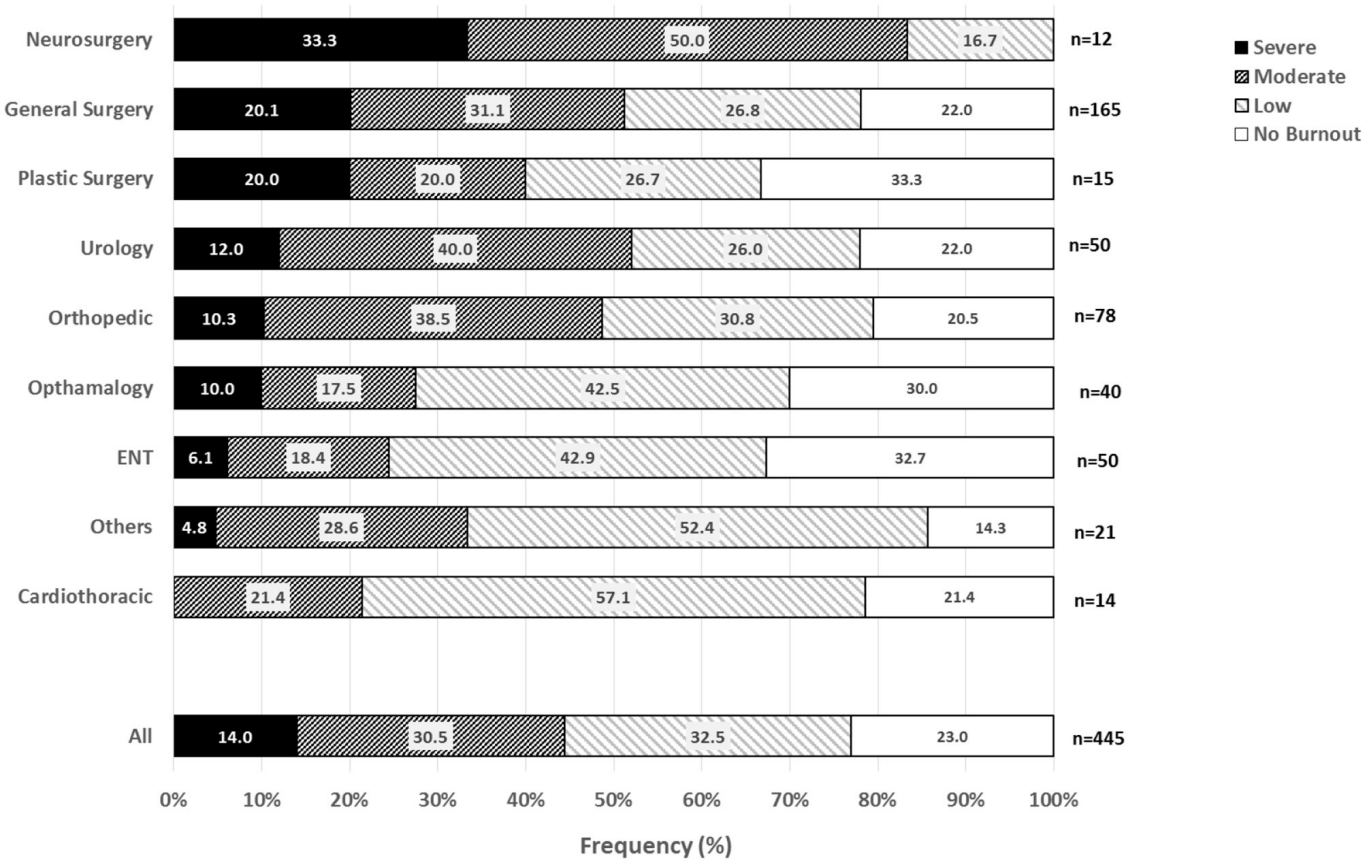
Distribution of burnout category, by surgical specialty.

The overall prevalence of burnout in this study was 76.9% [95% confidence interval, 72.9-80.7%]. The prevalence of severe burnout was 14.0% [11.0-17.5%] (Figure 2). For comparison with other studies which defined burnout as a score in the most extreme normative tertile of either the EE or DP subscales, the prevalence of burnout according to this alternative definition was 58.8% [54.0-63.4%].

Neurosurgeons had the highest prevalence of burnout (100% [73.5, 100.0]) and severe burnout (33.3% [9.9, 65.1%]). Vascular (100% [54.1, 100]) and transplant surgeons (100% [47.8, 100.0]) also had 100% burnout prevalence, however the sample sizes were small (*n* = 6 and *n* = 5, respectively), resulting in wider confidence intervals (data not shown in Figure 2). With regards to burnout and severe burnout, general surgeons, plastic surgeons, urologists, and orthopedic surgeons were next in prevalence, respectively. In contrast, ophthalmologic surgeons, ENT surgeons, cardiothoracic surgeons, and other surgical specialists had a generally lower prevalence of burnout and severe burnout.

After adjustment for potential confounding occupational factors, both burnout and severe burnout were associated with shorter career duration, younger age, and lower physician rank (Table 3). In addition, burnout alone was associated in the adjusted models with lower income and with more on-calls per month; whereas severe burnout was associated with current smoking, more working hours per day, and fewer weeks of vacation per year (Table 3). Results of the unadjusted models are provided in the online Supplementary Table 1).

**Table 3:** Adjusted associations of burnout and severe burnout with sociodemographic and occupational characteristics.

**Participant characteristics**	**Sub-group** ***n***	**Adjusted odds ratio of burnout^a^**			**Adjusted odds ratio of severe burnout^a^**		
		**AOR**	**[95% C.I.]**	***P***-**value**	**AOR**	**[95% C.I.]**	***P***-**value**
**Age** ^**a**^							
≤33	145	2.5	[1.4-4.3]	0.002	3.4	[1.4-8.4]	0.008
34-40	152	2.3	[1.3-4.3]	0.006	3.9	[1.6-9.5]	0.002
>40	148	1.0	Reference		1.0	Reference	
**Sex**							
Male	388	1.0	Reference		1.0	Reference	
Female	57	1.0	[0.5-2.1]	0.94	0.5	[0.2-1.3]	0.16
**Nationality**							
Kuwaiti	212	1.0	Reference		1.0	Reference	
Non-Kuwaiti	233	1.5	[0.8-2.6]	0.16	1.6	[0.8-3.3]	0.17
**Marital status**							
Single	96	1.0	Reference		1.0	Reference	
Married/divorced	349	0.9	[0.5-1.8]	0.76	0.8	[0.4-1.6]	0.51
**Income**							
<2,000	217	2.2	[1.2-4.1]	0.008	1.4	[0.6-3.1]	0.40
2,000-3,000	138	1.5	[0.8-2.7]	0.21	1.1	[0.5-2.6]	0.81
>3,000	90	1.0	Reference		1.0	Reference	
**Current smoking status**							
Non-smoker/Ex-Smoker	327	1.0	Reference		1.0	Reference	
Current Smoker	118	1.1	[0.6-2.0]	0.67	3.4	[1.9-6.2]	<0.001
**Physician rank**							
Assistant reg/registrar	277	2.1	[1.1-3.6]	0.01	2.9	[1.1-7.9]	0.03
Senior registrar	71	1.8	[0.8-3.8]	0.13	2.7	[0.8-8.5]	0.10
Specialist/consultant	97	1.0	Reference		1.0	Reference	
**Surgical specialty**							
Other specialties	280	1.0	Reference		1.0	Reference	
General surgery	165	1.1	[0.6, 1.8]	0.77	2.1	[1.2-3.9]	0.02
**Duration of surgical career**							
≤7 years	161	2.1	[1.1-3.8]	0.02	1.7	[0.7-4.1]	0.21
8-15 years	138	2.3	[1.3-4.1]	0.003	2.5	[1.1-5.6]	0.03
≥16 years	135	1.0	Reference		1.0	Reference	
**Working hours per day**							
<8 h	170	1.0	Reference		1.0	Reference	
8 h	151	1.2	[0.7-2.0]	0.62	2.1	[1.0-4.2]	0.051
>8 h	119	0.8	[0.5-1.5]	0.60	2.3	[1.1-5.0]	0.03
**On-calls per month**							
≤5 on-calls	103	1.0	Reference		1.0	Reference	
6-7 on-calls	125	2.4	[1.2-4.5]	0.008	2.0	[0.9-4.7]	0.11
8 on-calls	166	1.9	[1.1-3.5]	0.03	1/7	[0.7-4.0]	0.21
>8 on-calls	46	2.6	[1.0-6.2]	0.04	1.3	[0.4-4.3]	0.68
**Weeks of vacation/year**							
<4 weeks	102	1.6	[0.8-3.3]	0.20	3.0	[1.3-7.2]	0.01
4 weeks	195	1.0	[0.6-1.7]	0.91	2.5	[1.1-5.4]	0.03
>4 weeks	144	1.0	Reference		1.0	Reference	

a *All multivariable binary logistic regression models include general surgery (vs. all other specialties), career years, number of on-call/month, and number of vacation weeks/year, except that career years was not included in the models for age, rank, and income due to co-linearity. Before and after adjustment, location of surgical residency, weekly teaching hours, and private practice status were not significantly associated with either prevalence of burnout or severe burnout (data not shown)*.

Burnout and severe burnout were also associated with some of the self-identified sources of stress (Supplementary Table 2 in the online appendix). Burnout and severe burnout among those who indicated that being overloaded with cases was a source of stress were significantly higher (81.3%, *p* = 0.003 and 17.3%, *p* = 0.007 respectively). Additionally, having less time to spend with the family is another significant stressor that was reported by surgeons with burnout and severe burnout (80.8%, *p* = 0.003 and 16.6%, *p* = 0.016 respectively; online Supplementary Table 2).

The number of self-identified sources of stress were summed to produce a scale from 0 to 13. The median number of sources of stress in surgeons without any burnout was 5 [Interquartile Range 3-7], and in the burnout and severe burnout groups, it was 6 [3-8] and 7 [5-8.25], respectively (Kruskal-Wallis *p*-value = 0.002).

## Discussion

### Statement of Principal Findings

This cross-sectional study among 445 surgeons in Kuwait MOH hospitals found that about 3 out of 4 surgeons in Kuwait have burnout, defined as having extreme values on at least one of the three MBI subscales; with 1 out of 7 having severe burnout, as defined by extreme values on all three MBI subscales. Specialists in neurosurgery, general surgery, urology, and orthopedics were found to have very high levels of burnout (>75%) in Kuwait, whereas ophthalmology and ENT surgeons were among the lowest levels of burnout.

The most commonly reported sources of stress included case overload and having less time to spend with family. The factors significantly associated with burnout after adjustment are younger age, lower rank, lower income, shorter duration of surgical career, and higher number of on-call duty nights.

### Strengths and Weaknesses of the Study

A strength of this study is that surgeons were recruited from all Ministry of Health hospitals in Kuwait, improving the representativeness of the sample despite its use of convenience sampling. The study used the MBI, a validated measure of burnout used by many studies around the world. However, the MBI has not previously been validated in Arab populations. It is possible that interpretations of the questions may differ across cultures, and that completion of the questionnaire in English may have led to misinterpretation. However, this effect was likely minimized since all surgeons in Kuwait use English as the primary language in their professional work.

One weakness of the study was that sampling was conducted by convenience. It is possible that more burned-out surgeons may have declined to participate in the study, thus underestimating the prevalence of burnout. However, the non-response rate was only 9%, thus minimizing the impact of such a bias. Also, the prevalence we found was already very high, so if this bias were present, the true prevalence would be even higher. It seems unlikely that the more burned-out surgeons would be more likely to participate, although this is possible. A form of expectation bias could also be operative, with either under-reporting or over-reporting feelings related to burnout. There also could be under-reporting related to fear of retribution if superiors were to discover negative answers. However, confidentiality was assured in an effort to reduce such biases. The cross-sectional study design does not allow assessment of the direction of causality for associations. For example, the association of higher number of stressors and higher burnout could represent bi-directional causality.

### Strengths and Weaknesses in Relation to Other Studies

A recent review of burnout among healthcare professionals in Arab countries included 19 studies among physicians, nurses, and other healthcare workers in Arab countries (6), seven studies of which included only physicians and also used the MBI (6). Among the seven studies in physician populations, there were wide ranges of elevated EE sub-scores (29.5-81.0%), high DP sub-scores (15.7-64.3%), and low PA sub-scores (17.0-58.2%) (6). Our study findings fell directly in the middle of these ranges. Some of these differences could be due to the use of different versions of the MBI; use of varying cut-off points for the subscales; or less precise estimates owing to smaller sample sizes, as three of the studies had fewer than 100 participants (6). A strength of this study compared to other studies in the region is its relatively large sample size (*n* = 445), which represents almost half of the ~916 surgeons working in Kuwait's Ministry of Health hospitals.

Another strength of this study is the assessment of the association of burnout with certain characteristics and stressors. We found that after statistical adjustment, younger age, lower income, being a resident, more on-call nights per month, less time with family, and case overload were all associated with higher prevalence of burnout. These results were similar to the systematic review conducted by Galaiya et al., who reported that younger age, residency training, and increased workload were associated with burnout (16). While that systematic review reported that female sex and non-married status were associated with burnout, we found no associations with these two demographic factors. The associated factors we did find can inform Surgery Departments in the Kuwait MOH in the development of targeted programs and policies to reduce the high prevalence of burnout in this population. Further studies on the implementation of such interventions would also be warranted, to provide evidence of their effectiveness.

A large survey in the United States, which defined burnout alternatively as a high MBI EE score or DP score, enrolled 7,905 members of the American College of Surgeons, reporting a prevalence of 40% (11), a level much lower than that found in our study (58.8%) when using their definition. The prevalence reported by that study for the three components of burnout were 31.7% for a high EE score (compared to 44.7% in our study), 26% for a high DP score (compared to 43.1%), and 12.8% for a low PA score (compared to 47.2%) (11).

Our study is not able to provide a good explanation for the large difference in burnout prevalence compared with the US study, despite using the same instrument. This large difference could be true, or it could represent varying cultural perceptions of the questions or cultural differences in willingness to reveal burnout symptoms on a questionnaire. Most studies of burnout in physician populations have reported that surgeons had considerably higher levels of burnout compared to other medical specialties (2). However, the large US-based study of burnout among surgeons reported that the surgical specialties had a prevalence of burnout in the mid-range, compared to all medical specialties (11). In that study, Emergency Medicine, General Internal Medicine, and Neurology had the highest burnout prevalence. Such a comparison could not be made in this study as it did not include specialties other than surgery.

### Meaning of the Study

The prevalence of burnout among surgeons who participated in our study was 76.9% when defined by an extreme score on any of the three MBI axes, and 58.8% when defined by a high score on either the EE or DP axis. Such a high prevalence of burnout is very concerning. Surgery Departments should verify this high prevalence and explore explanations for it. Qualitative research methods using focus groups could provide detailed data regarding the environmental, personal, or cultural factors as potential reasons for the high prevalence. Organizational factors that may have led to such a high level of burnout could potentially include the hospital environment, management or leadership styles, heavy workload, inadequate pay, and horizontal and vertical communication within the medical staff (6). Personal factors could include emotional intelligence, mindfulness, extraversion, physical activity, mentorship, and support systems (16).

The consequences of burnout can include negative effects on patient care, the surgeon's mental well-being, and the healthcare system. With regard to effects on patient care, studies have reported associations of burnout with medical errors, lower quality of patient care, and decreased patient safety (3, 5, 11). One study reported that for every one point increase in the Maslach DP and EE subscale scores, the medical error report rate increased by 11 and 5%, respectively (17). Of note, over 40% of our participants were rated in the extreme range for both the DP and EE subscales, raising the possibility that medical errors could be resulting from such a high prevalence of burnout.

Studies in the general population have shown that burnout relates negatively to cognitive functioning and task performance *via* poorer working memory and increased cognitive failures (18, 19). Such negative cognitive effects are of great importance among surgeons, whose work require focus and attention for extended periods of time. Such effects could lead to errors, poor outcomes, and compromise of patient safety, particularly in long or consecutive surgical procedures or duty periods.

Given the high prevalence of burnout in our study, Occupational Health departments and administrative managers of hospitals in Kuwait should be made aware of these findings. Those in other countries in the region and elsewhere may also benefit by increasing awareness of this problem. Such administrators should develop tools to assess whether this high prevalence of burnout is affecting the quality of surgical care and patient safety before, during and after surgical procedures. Additionally, focused programs to address burnout in the surgical staff and procedures to mitigate the negative effects of burnout on patient care should also be developed and implemented.

Surgeons can also be personally affected by burnout through physical and emotional distress with an increased risk of headaches, sleep disturbances, hypertension, depression, and anxiety (1). A meta-analysis of 36 studies confirmed these findings showing that burnout is a predictor of 12 somatic diseases and negative health outcomes, among which are type 2 diabetes, coronary heart disease, headaches, respiratory diseases and mortality under the age of 45 years old (20). These negative personal health effects—not just the occupational effects on patient care—are important to consider when assessing the overall impact of burnout and its sequelae.

The meta-analysis above also found that insomnia, depression and hospitalization for mental disorders were also associated with burnout (20). However, there has been some discussion as to whether burnout is simply a different form of depression and/or anxiety, with significant overlaps (21). A meta-analysis of 67 papers on depression and burnout and 34 papers on anxiety and burnout found that while there were correlations between burnout and depression and also with anxiety, the analysis inferred that these three entities likely represent different, though associated, constructs (21). As such, burnout could be considered a risk factor for depression and anxiety (and perhaps vice-versa).

In addition to its physical and psychological consequences, burnout among surgeons is associated with the social stresses related to work-life conflicts (22), a relationship likely demonstrating bidirectional causation. Burnout at the workplace can place stress on family life, which in turn may cause family stresses that the surgeon brings back to the workplace in a spiral of work-life stresses and conflicts.

Regarding its effects on the healthcare system, physician burnout can lead to absenteeism from work, physician turnover, negative attitudes which can affect teamwork, and poor performance (23). When a trained physician loses time at work or leaves the profession, the large investment of the educational and healthcare system in training is, at least in part, lost. Consequences of physician burnout to the healthcare system therefore correspond to a high financial burden to the healthcare system. One can also theorize that after many years on the job, surgeons become acclimated to the demanding routine of the job.

This study identified some common sources of stress among surgeons, including life-work imbalance, case overload, verbal assault from patients, assignment of high-risk cases, failure to get deserved promotions, less time with family, and malpractice lawsuits. In particular, case overload and less time with family were significantly associated with burnout. The MOH, Occupational Health Departments and Departments of Surgery can use these findings as a guide to focus their initiatives toward reducing these stressors among surgeons, which may help to reduce burnout.

### Unanswered Questions and Future Research

Future studies, including qualitative studies, should explore the reasons why the prevalence of burnout is so high, why the range in the prevalence of burnout between subspecialties is so wide, and what interventions the surgeons themselves may implement to reduce burnout. Furthermore, the MOH should focus immediate attention on the surgical subspecialties that have the highest level of burnout to identify and mitigate the factors that contribute most to this finding. As a cross-sectional study, this study cannot assess these as causal risk factors for burnout, but they can be used as a basis for future cohort studies on burnout or studies of interventions designed to prevent burnout. These results can assist the MOH in developing and implementing policies that address the underlying causes of burnout, a syndrome which can detrimentally affect the quality of healthcare delivered and adversely affect the personal health and life of the surgeon (5, 6, 11). Programs that treat and prevent burnout among surgeons may be cost-effective and would have potential to improve the surgeons' health and quality of life and the quality of the patient care they deliver.

## Conclusion

In conclusion, the study of the prevalence of burnout amongst surgeons in Kuwait found a high prevalence of 76.9% with a significant association with common stressors. Levels of burnout varied among surgical subspecialties. This study aims to spread awareness about burnout and its effect on patients and surgeons as well as provide a stepping-stone for further research on the subject and a guide for occupational health initiatives.

## Data Availability Statement

The raw data supporting the conclusions of this article will be made available by the authors, without undue reservation.

## Ethics Statement

The studies involving human participants were reviewed and approved by the Kuwait University Health Sciences Center Ethics Committee for Undergraduate Research and the Kuwait Ministry of Health Standing Ethics Committee for the Coordination of Medical and Health Research on 12/24/2017 (MOH ID: 812). The participants provided their written informed consent to participate in this study.

## Author Contributions

JL: guarantor. IM: idea. AA, IM, RA, and OA: literature review. AA, IM, OA, SA, NT, and JL: planning. AA, IM, RA, OA, RD, AR, and SA: data collection and entry. AA, IM, OA, RD, NT, and JL: data analysis. AA, IM, RA, OA, RD, and JL: writing the paper. AA, IM, RA, OA, RD, AR, SA, NT, and JL: discussion. All authors contributed to the article and approved the submitted version.

## Conflict of Interest

The authors declare that the research was conducted in the absence of any commercial or financial relationships that could be construed as a potential conflict of interest.

## Publisher's Note

All claims expressed in this article are solely those of the authors and do not necessarily represent those of their affiliated organizations, or those of the publisher, the editors and the reviewers. Any product that may be evaluated in this article, or claim that may be made by its manufacturer, is not guaranteed or endorsed by the publisher.

## Abbreviations

CI: Confidence Interval
DP: Depersonalization
EE: Emotional Exhaustion
MBI: Maslach Burnout Inventory
MOH: Ministry of Health
PA: Personal accomplishment
SD: Standard deviation.
