# Supplementary material for: The Prevalence of Burnout and Its Associated Factors Among Surgical Specialists in Kuwait Ministry of Health Hospitals
Source: Front Public Health. 2022 Jan 31;10:679834. doi: 10.3389/fpubh.2022.679834 (PMC8841660; doi:10.3389/fpubh.2022.679834)
Supplement: Supplementary file 1 [file Table_1.docx]

**Supplementary File**

Contents:

Online Appendix tables

- Table A: un-adjusted associations of Burnout and Severe Burnout with sociodemographic characteristics (Part 1) and occupational characteristics (Part 2).
- Table B: un-adjusted association between Burnout/severe Burnout and potential sources of stress

| **Table A:** associations of Burnout and Severe Burnout with sociodemographic characteristics | | | | | | | | |
| --- | --- | --- | --- | --- | --- | --- | --- | --- |
| **Sociodemographic Characteristics** | | **Prevalence of Burnout** | | **p-value** |  | **Prevalence of Severe Burnout** | | **p-value** |
|  |  | **n** | **(%)** |  |  | **n** | **(%)** |  |
| **Overall** | | **342** | **(76.9)** |  |  | **62** | **(14.0)** |  |
|  | |  |  |  |  |  |  |  |
| **Age** | |  |  | 0.001 |  |  |  | <0.001 |
| ≤33 | | 119 | (82.1) |  |  | 28 | (19.3) |  |
| 34-40 | | 125 | (82.2) |  |  | 27 | (17.8) |  |
| >40 | | 98 | (66.2) |  |  | 7 | (4.7) |  |
|  | |  |  |  |  |  |  |  |
| **Gender** | |  |  | 0.79 |  |  |  | 0.23 |
| Male | | 299 | (77.1) |  |  | 57 | (14.7) |  |
| Female | | 43 | (75.4) |  |  | 5 | (8.8) |  |
|  | |  |  |  |  |  |  |  |
| **Nationality** | |  |  | 0.38 |  |  |  | 0.90 |
| Kuwaiti | | 159 | (75.0) |  |  | 30 | (14.2) |  |
| Non-Kuwaiti | | 183 | (78.5) |  |  | 32 | (13.7) |  |
|  |  |  |  |  |  |  |  |  |
|  | |  |  |  |  |  |  |  |
| **Marital status** | |  |  | 0.28 |  |  |  | 0.035 |
| Single | | 84 | (80.8) |  |  | 21 | (20.2) |  |
| Married | | 258 | (75.7) |  |  | 41 | (12.0) |  |
|  | |  |  |  |  |  |  |  |
| **Income** | |  |  | 0.002 |  |  |  | 0.20 |
| <2000 | | 179 | (82.5) |  |  | 35 | (16.1) |  |
| 2001-3000 | | 103 | (74.6) |  |  | 17 | (12.3) |  |
| >3000 | | 60 | (66.7) |  |  | 10 | (11.1) |  |
|  | |  |  |  |  |  |  |  |
| **Smoking status** | |  |  | 0.27 |  |  |  | <0.001 |
| Non-smoker | | 247 | (75.5) |  |  | 31 | (9.5) |  |
| Smoker | | 95 | (80.5) |  |  | 31 | (26.3) |  |
|  | |  |  |  |  |  |  |  |

| **Table A**: associations of Burnout and Severe Burnout with occupational characteristics | | | | | | | |
| --- | --- | --- | --- | --- | --- | --- | --- |
| **Occupational Characteristics** | **Prevalence of Burnout** | | **p-value** |  | **Prevalence of Severe Burnout** | | **p-value** |
|  | **n** | **(%)** |  |  | **n** | **(%)** |  |
| **Physician Rank** |  |  | 0.007 |  |  |  | 0.005 |
| Assistant Reg/Registrar | 223 | (80.5) |  |  | 47 | (17.0) |  |
| Senior Registrar | 54 | (76.1) |  |  | 10 | (14.1) |  |
| Specialist/Consultant | 65 | (67.0) |  |  | 5 | (5.2) |  |
|  |  |  |  |  |  |  |  |
| **Surgical specialty** |  |  |  |  |  |  | 0.29^a^ |
| Neurosurgery | 12 | (100.0) |  |  | 4 | (33.0) |  |
| Vascular Surgery | 6 | (100.0) |  |  | 0 | (0.0) |  |
| Transplant Surgery | 5 | (100.0) |  |  | 1 | (20.0) |  |
| Orthopedic Surgery | 62 | (79.5) |  |  | 8 | (10.3) |  |
| Cardiothoracic Surgery | 11 | (78.6) |  |  | 0 | (0.0) |  |
| General Surgery | 129 | (78.2) |  |  | 33 | (20.0) |  |
| Urology | 39 | (78.0) |  |  | 6 | (12.0) |  |
| Oncology Surgery | 6 | (75.0) |  |  | 0 | (0.0) |  |
| Ophthalmologic Surgery | 28 | (70.0) |  |  | 4 | (10.0) |  |
| Plastic Surgery | 10 | (66.7) |  |  | 3 | (20.0) |  |
| ENT Surgery | 33 | (66.0) |  |  | 3 | (6.0) |  |
| Maxillofacial Surgery | 1 | (50.0) |  |  | 0 | (0.0) |  |
|  |  |  |  |  |  |  |  |
| **Surgical Residency location** |  |  | 0.52 |  |  |  | 0.15^b^ |
| Kuwait | 115 | (74.7) |  |  | 25 | (16.2) |  |
| Arab countries and Asia | 170 | (78.0) |  |  | 28 | (12.8) |  |
| US/Canada/Australia/EU | 34 | (70.8) |  |  | 4 | (8.3) |  |
|  |  |  |  |  |  |  |  |
| **Duration of Surgical Career** |  |  | 0.003 |  |  |  | 0.015 |
| ≤ 7 years | 114 | (80.9) |  |  | 24 | (17.0) |  |
| 8-15 years | 130 | (82.3) |  |  | 27 | (17.1) |  |
| ≥ 16 years | 90 | (66.7) |  |  | 9 | (6.7) |  |
|  |  |  |  |  |  |  |  |
| **Working hours per day** |  |  | 0.74 |  |  |  | 0.028 |
| < 8 hours | 129 | (75.9) |  |  | 14 | (8.2) |  |
| 8 hours | 120 | (79.5) |  |  | 25 | (16.6) |  |
| 9-10 hours | 59 | (73.8) |  |  | 17 | (21.3) |  |
| > 10 hours | 31 | (79.5) |  |  | 5 | (12.8) |  |
|  |  |  |  |  |  |  |  |
| **Teaching hours per week** |  |  | 0.78 |  |  |  | 0.85 |
| 0 hours | 130 | (78.3) |  |  | 24 | (14.5) |  |
| 1-5 hours | 123 | (75.9) |  |  | 21 | (13.0) |  |
| > 5 hours | 88 | (79.3) |  |  | 17 | (15.3) |  |
|  |  |  |  |  |  |  |  |
| **Number of On-calls per Month** |  |  | 0.011 |  |  |  | 0.30 |
| ≤ 5 on-calls | 68 | (65.4) |  |  | 9 | (8.7) |  |
| 6-7 on-calls | 103 | (82.4) |  |  | 20 | (16.0) |  |
| 8 on-calls | 131 | (78.9) |  |  | 26 | (15.7) |  |
| > 8 on-calls | 38 | (82.6) |  |  | 5 | (10.9) |  |
|  |  |  |  |  |  |  |  |
| **Number of weeks of vacation/year** |  |  | 0.21 |  |  |  | 0.006 ^b^ |
| < 4 weeks | 85 | (83.3) |  |  | 20 | (19.6) |  |
| 4 weeks | 147 | (75.4) |  |  | 31 | (15.9) |  |
| > 4 weeks | 108 | (74.5) |  |  | 11 | (7.6) |  |
|  |  |  |  |  |  |  |  |
| **Practice in Private sector** |  |  | 0.49 |  |  |  | 0.25 |
| No | 294 | (77.4) |  |  | 56 | (14.7) |  |
| Yes | 47 | (73.4) |  |  | 6 | (9.4) |  |

^a^ P-value generated using a Monte-Carlo simulation of 10,000 iterations, approximating the Fisher’s Exact test.

^b^ P-value for linear trend

| **Table B:** the association between Burnout/severe Burnout and potential sources of stress | | | | | | | |
| --- | --- | --- | --- | --- | --- | --- | --- |
| **Potential sources of stress** | **Prevalence of Burnout** | | **p-value** |  | **Prevalence of Severe Burnout** | | **p-value** |
|  | **n** | **(%)** |  |  | **n** | **(%)** |  |
|  |  |  |  |  |  |  |  |
| **Miscommunication with nurses in the OT** |  |  | 0.21 |  |  |  | 0.46 |
| Not stressful | 251 | (78.4) |  |  | 15 | (12.0) |  |
| Stressful | 91 | (72.8) |  |  | 47 | (14.7) |  |
| **Miscommunication w surgical colleagues** |  |  | 0.36 |  |  |  | 0.042 |
| Not stressful | 212 | (75.4) |  |  | 32 | (11.4) |  |
| Stressful | 130 | (79.3) |  |  | 30 | (18.3) |  |
| **Being passed over for interesting cases** |  |  | 0.82 |  |  |  | 0.004 |
| Not stressful | 215 | (76.5) |  |  | 29 | (10.3) |  |
| Stressful | 127 | (77.4) |  |  | 33 | (20.1) |  |
| **Being assigned high-risk cases** |  |  | 0.13 |  |  |  | 0.021 |
| Not stressful | 121 | (72.9) |  |  | 15 | (9.0) |  |
| Stressful | 221 | (79.2) |  |  | 47 | (16.8) |  |
| **Being overloaded with cases** |  |  | 0.003 |  |  |  | 0.007 |
| Not stressful | 111 | (68.9) |  |  | 13 | (8.1) |  |
| Stressful | 231 | (81.3) |  |  | 49 | (17.3) |  |
| **Not being assigned enough cases** |  |  | 0.80 |  |  |  | 0.84 |
| Not stressful | 238 | (76.5) |  |  | 44 | (14.1) |  |
| Stressful | 104 | (77.6) |  |  | 18 | (13.4) |  |
| **Inadequate supervision by seniors** |  |  | 0.20 |  |  |  | 0.035 |
| Not stressful | 212 | (74.9) |  |  | 32 | (11.3) |  |
| Stressful | 130 | (80.2) |  |  | 30 | (18.5) |  |
| **Residency exams** |  |  | 0.58 |  |  |  | 0.011 |
| Not stressful | 192 | (75.9) |  |  | 26 | (10.3) |  |
| Stressful | 150 | (78.1) |  |  | 36 | (18.8) |  |
| **Failure to get deserved promotions** |  |  | 0.45 |  |  |  | 0.068 |
| Not stressful | 132 | (75.0) |  |  | 18 | (10.2) |  |
| Stressful | 210 | (78.1) |  |  | 44 | (16.4) |  |
| **Malpractice lawsuits** |  |  | 0.76 |  |  |  | 0.048 |
| Not stressful | 137 | (76.1) |  |  | 18 | (10.0) |  |
| Stressful | 205 | (77.4) |  |  | 44 | (16.6) |  |
| **Telephone calls from patients** |  |  | 0.001 |  |  |  | 0.90 |
| Not stressful | 182 | (71.4) |  |  | 36 | (14.1) |  |
| Stressful | 160 | (84.2) |  |  | 26 | (13.7) |  |
| **Verbal assault from patients or families** |  |  | 0.99 |  |  |  | 0.56 |
| Not stressful | 116 | (76.8) |  |  | 19 | (12.6) |  |
| Stressful | 226 | (76.9) |  |  | 43 | (14.6) |  |
| **Having less time to spend with family** |  |  | 0.003 |  |  |  | 0.016 |
| Not stressful | 93 | (67.9) |  |  | 11 | (8.0) |  |
| Stressful | 249 | (80.8) |  |  | 51 | (16.6) |  |
